# The impact of ketamine on cognitive outcomes in geriatric anesthesia: a comprehensive review

**DOI:** 10.3389/fpsyt.2025.1594730

**Published:** 2025-07-30

**Authors:** Shuyong You, Zhaohui Li

**Affiliations:** ^1^ Department of Anesthesiology, Luxian People’s Hospital, Luzhou, Sichuan, China; ^2^ Department of Anesthesiology, The Second People’s Hospital of Luxian County, Luzhou, Sichuan, China

**Keywords:** ketamine, geriatric anesthesia, postoperative cognitive dysfunction, post-operative delirium, neuroprotection, NMDA receptor antagonist

## Abstract

**Background:**

Ketamine, a dissociative anesthetic with N-methyl-D-aspartate (NMDA) receptor blockade, has become increasingly popular in geriatric anesthesia because of its hemodynamic stability, lack of respiratory depression, and possible neuroprotective properties. Yet, its effect on cognitive function in elderly surgical patients is unknown. Postoperative cognitive dysfunction (POCD) and postoperative delirium (POD) are frequent complications in elderly surgical patients, resulting in longer hospital stays, higher healthcare costs, and long-term cognitive impairment. Although there is some evidence to support ketamine in decreasing neuroinflammation and maintaining cognitive function, others describe high risks of delirium and hallucination, especially at higher doses.

**Methods:**

This review assessed the existing literature on ketamine’s impact on cognitive outcomes in older anesthesia. A comprehensive review of randomized controlled trials (RCTs was performed, assessing ketamine’s potential to prevent or worsen POCD and POD.

**Results:**

Results show that low-dose ketamine (0.3–0.5 mg/kg) is neuroprotective and decreases the rate of cognitive dysfunction in certain patients. Nevertheless, findings continue to be at odds because study design, population of patients, dosing schedules, and measure of cognition may differ. Secondly, the weighting of ketamine’s neuroprotective and neurotoxic effects is dose-dependent with larger doses inducing unwanted neuropsychiatric impacts.

**Conclusion:**

In light of these divergent results, additional large-scale, multicenter RCTs are needed to establish optimal dosing regimens and to identify elderly patient subgroups that could be treated safely with ketamine to avoid cognitive complications. Multimodal techniques of anesthesia and long-term cognitive outcomes will also need to be studied in future studies to further delineate ketamine’s definitive place in geriatric anesthesia.

## 1 Introduction

Over the past few years, there has been a rapid increase in the use of ketamine in geriatric anesthesia because of its special properties. Developed as a dissociative anesthetic, the ketamine primarily functions through N-methyl-D-aspartate (NMDA) receptor antagonism, which leads to the modulation of glutamatergic transmission and results in anesthetic and analgesic activities (1). Due to its stability in hemodynamics, minimal respiratory depression, and possible neuroprotective features, ketamine has become the preferred choice for elderly surgical patients with multiple comorbidities for whom traditional anesthetic agents are usually contraindicated (2).

Surgery in the elderly poses a significant risk for both delirium and cognitive dysfunction, thus cognitive function is a major issue that needs to be considered. Postoperative delirium (POD) represents an acute cognitive impairment that occurs during the postoperative period, and postoperative cognitive dysfunction (POCD) represents more prolonged cognitive problems in memory and executive and attention functions, which may persist from weeks to months (3). These neurocognitive disorders are both related to higher morbidity and longer hospitalizations and increased healthcare costs and also cause long-term cognitive deterioration (4). With an aging world population, reducing perioperative neurocognitive dysfunction is now considered an important anesthetic practice (5).

The effect of ketamine on cognitive outcomes is not clearly defined by its potential neuroprotective activity. The literature presents conflicting evidence regarding how ketamine affects neuroinflammation and long-term cognitive function, with some evidence showing protective effects but other studies demonstrating increased risks of hallucinations and delirium, and long-term cognitive impairment (6). Additionally, the dose-response relationships present challenges in clinical practice because the neurocognitive effects of low-dose ketamine differ from those of high-dose ketamine administration (7). This review seeks to provide clinicians with evidence-based insights into the potential effect of ketamine on cognitive outcomes in geriatric anesthesia.

We conducted a comprehensive search of peer-reviewed literature focusing on randomized controlled trials (RCTs) published up to May 2025. We assessed studies evaluating ketamine’s cognitive effects in patients aged 55 and older who received ketamine anesthesia, and outcomes including POCD, POD, long-term cognitive function, and neuroprotection were reported. We aimed to provide a balanced view of ketamine’s risks and benefits in geriatric cognitive outcomes, with a focus on clinical applicability.

## 2 Discovery and initial development of ketamine

Ketamine was originally synthesized in 1962 by chemist Calvin L. Stevens at Parke-Davis Laboratories as a search for a substitute for phencyclidine (PCP), a highly potent anesthetic with long-lasting hallucinogenic activity and severe neurotoxicity (8). PCP, initially developed in the 1950s, was a very potent anesthetic but frequently produced postoperative agitation and confusion of a severe nature, and therefore was of limited clinical value. In comparison, ketamine was a more short-acting dissociative anesthetic that retained PCP’s anesthetic advantage but drastically minimized its unwanted side effects (9). Human testing of ketamine began in 1964 on inmates at Jackson Prison in Michigan. The findings showed that ketamine could produce anesthesia quickly with less depression of respiration and cardiovascular stability. This characteristic made it a serious candidate for surgical anesthesia and emergency medicine (8). It was soon approved by the U.S. Food and Drug Administration (FDA) in 1970 under the brand name Ketalar as a general anesthetic (10). The new mode of action of ketamine as an antagonist at N-methyl-D-aspartate (NMDA) receptors places it apart from the other anesthetics. Unlike standard inhalational or intravenous anesthetics, whose primary action in producing unconsciousness is on the gamma-aminobutyric acid (GABA) receptors, ketamine suppresses excitatory glutamate neurotransmission with dissociative anesthesia. This enables patients to stay in a state of cataleptic trance with intact airway reflexes, spontaneous respiration, and relative hemodynamic stability, and therefore it is an agent of preference for patients at risk of hypotension or respiratory depression (11).

## 3 Early applications of ketamine in clinical anesthesia

Following its approval, ketamine was quickly adopted in many different medical environments, especially in warfare anesthesia, emergency medicine, and pediatric anesthesia. Due to its effective analgesia and anesthesia without sacrificing respiratory status, ketamine was used heavily by the U.S. military in Vietnam. It became the drug of choice for administration in field settings where supplies were limited and rapid anesthetic induction was desired (8). Ketamine was also highly used for sedation for procedures, especially in trauma patients, burn patients, and for rapid sequence intubation. It was the only anesthetic that did not involve advanced airway management, so it was a safe one to use away from the operating room. Moreover, the drug was also preferentially used in children who were undergoing minor surgical procedures since it was an effective anesthetic without the need for intubation (12). However, ketamine’s initial use was also clouded by complaints of its psychotomimetic side effects, such as hallucinations, dissociation, and agitation, in adult patients. These side effects, also referred to as “emergence delirium,” resulted in it being disfavored with the introduction of newer drugs such as propofol and midazolam in the 1980s and 1990s (13).

## 4 Mechanism of action of ketamine

The primary mechanism of action of ketamine in the brain is its blockade of NMDA receptors, which inhibits the excitatory neurotransmitter glutamate from acting on its receptor. This reduces the entry of calcium ions into neurons, thus preventing excessive neuronal firing that will lead to excitotoxicity and neuronal death. Apart from NMDA receptors, ketamine also exerts action on other receptors, including opioid, muscarinic, and monoamine receptors, which are responsible for its complex pharmacological effects. These interactions are believed to be responsible for its anesthetic, an analgesic, and possibly neuroprotective effects (14).

Cognitively, the NMDA blockade by ketamine could have dramatic effects. Glutamate excitotoxicity has been implicated in the pathophysiology of several neurodegenerative diseases, such as Alzheimer’s disease. Thus, ketamine’s capacity to modulate this pathway could be neuroprotective, especially in the elderly, whose brains are generally more susceptible to neurodegeneration (14). But this antagonism is also linked with dissociative and psychotomimetic effects, such as hallucinations, disorientation, and cognitive impairment, which are more marked at higher doses (15).

Notably, ketamine also increases neuroplasticity, which has potential therapeutic application for cognitive illnesses such as depression and Alzheimer’s disease. Research has indicated that subanesthetic doses of ketamine release brain-derived neurotrophic factor (BDNF), which plays a role in synaptic plasticity and neuronal growth. This action can be especially useful in reversing cognitive impairment, although the mechanisms are still the subject of ongoing research (16).

## 5 Methods

We conducted a comprehensive search in three databases, including PubMed, Web of Science, and Scopus. This search was further supplemented by a manual search to ensure completeness (including Google Scholar and backward citation searching of relevant studies). Detailed keywords and search strategies for each database are available in **Supplementary Tables S1** and **S2**. After removing duplicates, two independent reviewers screened the titles and abstracts of the identified records. The studies included in this review met the following criteria: (1) randomized controlled trials (RCTs) (2) published in peer-reviewed journals in the English language, (3) involving patients older than 50 years, and (4) reporting data on post-operative delirium or post-operative cognitive dysfunction after anesthesia with ketamine. Relevant data were extracted by two authors. This data collection followed a prepared checklist that included individual patient details, such as first author, year, country, sample size, inclusion criteria, prescribed time and dosage of ketamine, comparison group, research design, instrument for cognitive assessments, results, and summary of cognitive outcomes.

## 6 The geriatric transition to anesthesia: reexamining the role of ketamine

Though ketamine was first applied more frequently in young patients, its application in geriatric anesthesia started to be explored during the late 1990s and early 2000s when researchers were looking for anesthetic drugs that would minimize POCD and POD in elderly patients. Elderly patients are especially susceptible to cognitive dysfunction from anesthesia, and this can result in prolonged hospitalization, greater morbidity, and prolonged cognitive impairment (17). Aging is linked with enhanced vulnerability to neuroinflammation, oxidative stress, and neurodegeneration, all of which are implicated in POCD and POD. Conventional anesthetics like inhalational agents (e.g., sevoflurane, isoflurane) and benzodiazepines (e.g., midazolam) have been linked with enhanced neurotoxicity and enhanced postoperative cognitive impairment. Conversely, ketamine’s potential to modulate glutamate transmission and promote synaptic plasticity hinted at a possible neuroprotective effect in elderly patients. Another certain benefit of ketamine in geriatric age anesthesia is its cardiovascular stability. Most elderly patients already have pre-existing cardiovascular disease, for which they are predisposed under general anesthesia because of hypotension and ischemic complications. In comparison to propofol or volatile agents, which always cause deep blood pressure reduction, ketamine maintains sympathetic tone, which minimizes the risks of perioperative hemodynamic instability (18). Given the concern about the administration of opioids to geriatric patients, i.e., increased risk of respiratory depression, constipation, and delirium, the analgesic effect and opioid-sparing action of ketamine made it a contender for being included as an adjuvant in multimodal pain control plans (19).

## 7 Pharmacokinetics and pharmacodynamics of ketamine in elderly patients

The pharmacokinetics of ketamine - drug distribution, metabolism, and excretion, as well as drug absorption by the body - are also quite different in older patients from those of younger groups. Impairment of liver and kidney function with advancing age and redistribution of fat and lean body mass influence the pharmacodynamics and action of the drug (20).

In the elderly, the hepatic metabolism of ketamine by the cytochrome P450 enzymes is generally slower, with resultant prolonged plasma levels of the drug and heightened risk of side effects. Additionally, renal changes can affect ketamine’s metabolites. Even though the clinical impact of these pharmacokinetic changes is questionable, elderly patients would receive lower or modified doses of ketamine to prevent undue sedation or cognitive impairment (21).

The pharmacodynamic sensitivity of elderly patients to the actions of ketamine is yet another factor of great significance. Aging results in a lowered threshold for anesthetic action, so that elderly patients require smaller doses of anesthetics to induce the same clinical effects. The increased sensitivity is thought to be due to changes in brain receptor density, neurotransmitter activity, and reduced cerebral blood flow. These are the same factors involved in increasing the risk of acute cognitive dysfunction and long-term cognitive impairment (22).

## 8 The revival of ketamine in geriatric anesthesia

In the early 2000s, a few randomized controlled trials (RCTs) considered the impact of ketamine on postoperative cognitive function in elderly patients. The intraoperative low-dose use of ketamine was found by a study to be related to reduced POD in older surgical patients. The mechanism was supposed to be based on its anti-inflammatory effect as well as a blockade of neurotoxic glutamate excitotoxicity. Although some trials had previously reported ketamine to decrease the occurrence of POCD, others observed no difference compared to placebo. Outcome heterogeneity was most likely explained by heterogeneity of dose, timing, and patient populations among studies. To avoid the risk of psychotomimetic side effects, the researchers started seeking the application of sub-anesthetic doses (0.3–0.5 mg/kg) or continuous infusions of low doses rather than bolus administrations. These approaches were intended to take advantage of the neuroprotective and analgesic properties of ketamine without risking hallucinations and agitation in the patient (23).

## 9 Ketamine’s dual role: neuroprotection vs. neurotoxicity

The balance between ketamine’s neuroprotective and neurotoxic actions remains under investigation. On the positive side, ketamine NMDA receptor blockade has been shown to safeguard the brain against excitotoxicity caused by ischemic damage or neurodegenerative disorders. Its capacity to suppress inflammatory cytokines and promote synaptic plasticity indicates that it may have a positive function in disorders such as Alzheimer’s disease, which are characterized by neuroinflammation and synaptic dysfunction (24, 25).

Conversely, high-dose or long-term ketamine administration has also been associated with neurotoxicity and cognitive function impairments, especially when administered in anesthesia. Data from both human and animal experiments have indicated that chronic ketamine exposure is potentially associated with memory loss, neurodegeneration, and cognitive impairment (26). These effects are likely a result of dose-dependent effects, with the protective effects being at low doses and larger doses having the potential to enhance cognitive impairment by interfering with normal neuronal functions as well as neuroinflammation promotion.

In addition, ketamine’s dissociative acute administration effects, e.g., hallucinations, confusion, and withdrawal from emotion—are common among patients at high doses. These effects can become particularly disruptive in the normal function of elderly patients and result in POD or exacerbating pre-existing cognitive impairments (27).

The biphasic nature of the action of ketamine requires extreme caution in regard to dose and timing of administration in the use of the drug in geriatric anesthesia. Though it has promise as a neuroprotective agent, use must be weighed against the hazard of delirium, psychotic behavior, and long-term impairment of cognitive function, especially with established cognitive impairment patients (28).

## 10 Postoperative cognitive effects of ketamine in the elderly population

**Figure 1:**
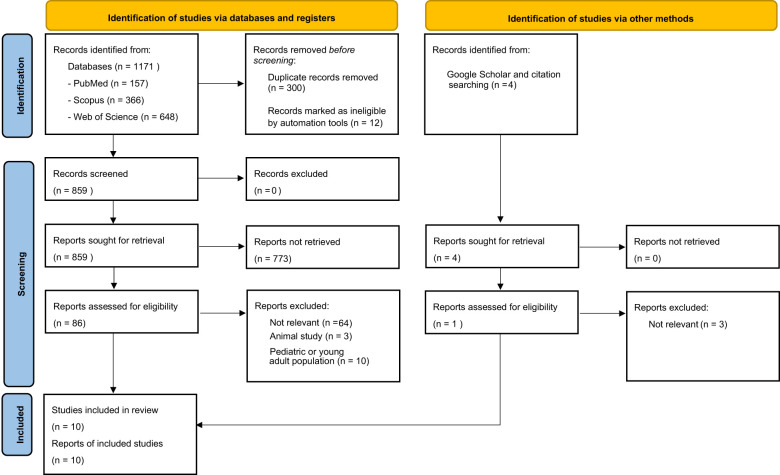
PRISMA flow diagram illustrating the study selection process for the literature review including identification, screening, eligibility and inclusion of studies.

Through our systematic search, 1171 records were identified (PubMed: n=157, Web of Science: n=366, and Scopus: n=648). After removing duplicate and ineligible publications (n=312), 859 studies were screened by title and abstract. For the next step, 86 studies were assessed for eligibility according to their corresponding full text. We also assessed 4 additional studies, which were identified using Google Scholar and backward citation searching of relevant studies. Finally, 10 studies were included in this systematic review. **Figure 1** presents the flow diagram of the included and excluded articles in our review according to PRISMA.

Numerous studies have investigated the impact of ketamine on postoperative cognitive function in elderly patients undergoing various types of surgeries. The findings, however, remain inconclusive, with some studies suggesting a protective effect, while others report no significant differences between ketamine and control groups (**Table 1**).

**Table 1:** Clinical trials that assessed cognitive outcomes after anesthesia using ketamine in elderly patients.

Author, year, country	Sample size and inclusion criteria	Prescribed time and dosage of Ketamine	Comparison group	Research design and instrument	Results	Summary of cognitive outcomes
Wittwer (34), 2023, USA	N = 52 (n = 25 in the ketamine group and n = 24 in the propofol group) + age > 70 years + surgery involving more than one heart valve, redo sternotomy procedures, or combined valvular and CABG	Induction + 1–2 mg/kg ketamine intravenously	0.5–1 mg/kg propofol	Prospective randomized study; CAM	There was no difference in the incidence of POD between the two groups (P = 0.23). Ketamine has no advantage or disadvantage in the induction of anesthesia compared to propofol in the induction of delirium.	No significant difference
Siripoonyothai (36), 2021, Thailand	N = 82 (n = 32 in each group) + age > 65 years + CABG and valvular surgery	During CPB +1 mg/kg/h ketamine	During CPB + 1.5–6 mg/kg/h propofol	Randomized controlled trial; CAM-ICU	The risk of POD was higher in the Propofol group. No occurrence of nightmares and hallucinations up to 24 hours after the operation in both groups.	Protective effect for POD
Avidan (27), 2017, USA	N = 672 (n = 222 in the placebo group, n = 227 in the 0.5 mg/kg ketamine group, and n = 223 in the 1.0 mg/kg ketamine group) + age > 60 years + undergoing major cardiac (coronary artery bypass graft (CABG) or valve replacement) and noncardiac surgery under general anesthesia	After induction of anesthesia and before surgical incision+ 0.5 or 1.0 mg/kg ketamine	Equivalent volume of normal saline	Multicenter, international randomized trial; CAM/CAM-ICU	Time to delirium onset, duration of delirium, and delirium severity did not differ significantly between the three groups over postoperative days 1–3. There was no difference in the incidence of POD between the groups (P = 0.80). With increasing ketamine dose, more patients reported hallucinations (P= 0.01) and nightmares (P = 0.03).	No significant difference
Soltanzadeh (33), 2013, Iran	N = 40 (n = 20 each group) + age > 55 years + CABG on Pump	0.5 mg/kg ketamine IV before sternotomy and repeated the same dose during surgery	The same volume of normal saline	Double-blind clinical trial; SAS	The reduction of agitation in the ketamine group in the first 24 hours was not statistically significant) P > 0.05). Within 72 hours after the operation, the agitation of the patients in the ketamine group was less than the placebo (P < 0.05). + The hemodynamics of the patients did not differ between the two groups (P < 0.05).	Possible reduction in agitation in the ketamine group
Hudetz (30), 2009, USA	N= 58 (n=29 in ketamine group, n=29 in placebo group) + age between 55 to 84 years + elective coronary artery bypass graft surgery or valve replacement/repair procedures with CPB	intravenous single dose of ketamine 0.5 mg/kg	An equal volume of placebo (0.9% saline)	A prospective randomized study, Intensive Care Delirium Screening Checklist	The incidence of postoperative delirium was significantly lower (p =0.01, Fisher exact test) in patients receiving ketamine (1/29) compared with placebo (9/29). The odds of developing postoperative delirium were approximately 13 times greater for patients receiving placebo treatment compared with patients receiving ketamine treatment (odds ratio= 12.6; 95% confidence interval, 1.48-107.5; logistic regression).	Protective effect for POD
Hudetz (29), 2009, USA	N= 52 (n=26 in ketamine group, n=26 in placebo group, n=26 in non-surgical control group) + age ≥ 55 years + cardiac surgery	intravenous bolus of ketamine 0.5 mg/kg	an equal volume of placebo (0.9% saline)	Randomized controlled trial; a battery of neurocognitive tests	Cognitive performance after surgery decreased by at least 2 SDs (z-score of 1.96) in 21 patients in the placebo group and only in seven patients in the ketamine group compared with the nonsurgical controls (P<001).	Fewer patients experienced cognitive decline in the ketamine group
Lee (35), 2015, South Korea	N=51 (n=26 in control group, n=25 in ketamine group) + age > 60 years + Orthopedic Surgery	a total of 3 mL mixed with 0.9% normal saline and 0.5 mg/kg ketamine	a total of 3 mL mixed with 0.9% normal saline	Randomized controlled trial; TMT, MMSE, and DST, digit substitution test.	There were no significant differences in the mini-mental status examination (P = 0.19), trail-making test (P = 0.08), and digit substitution test scores (P = 0.28) between the two groups throughout the time.	No significant differences
Rascón-Martínez (37), 2016, Mexico	N=65, (n=33 in ketamine group, n=32 in control group), age > 60 years+ Ophthalmic Surgery	0.3 mg/kg in a physiologic solution at 0.9% (250 mL)	physiologic solution at 0.9%	Randomized controlled trial; SPMSQ	After surgery, an increased number of patients in the ketamine group performed within the normal range (n = 28, 84.8%; P = 0.03), whereas the percentage of patients in the control group with a normal cognitive performance remained almost unchanged (n = 24, 75%; P = 0.62).	Improved cognitive scores post-op in ketamine group
Oriby (31), 2023, Egypt	N=90 (Control group (n=30), Ketamine group (n=30), Dexmedetomidine group (n=30)) + age between 65–85 years + cataract surgery	ketamine by continuous infusion (0.3 mg/kg/h)	Normal saline and 0.1 ml/kg/h or Dexmedetomidine	Randomized controlled trial; a battery of neurocognitive tests	In comparison with control group, ketamine and dexmedetomidine groups exhibited a significant decline in number of patients who developed POCD (P < 0.0001),	Significant reduction in POCD incidence in the ketamine group
Zhang (32), 2013, China	N= 120 (ketamine group (n=30), dexmedetomidine group (n=30), ketamine + dexmedetomidine (n=30) group and control group (n=30)) + age ≥ 60 years + selective orthopedic surgery	0.5 mg/kg ketamine intravenous injection	dexmedetomidine 1 μg/kg followed by 0.5 μg/kg/h infusion or saline	Randomized controlled trial; NM	Compared with control group, the incidence of POCD in ketamine group at 1, 7 d after operation was significantly decreased (P < 0.05), the incidence of POCD in dexmedetomidine group and ketamine + dexmedetomidine group at 1, 7 d after operation had no significant differences (P> 0.05)	Protective effect on POCD

CABG, Coronary artery bypass grafting; CAM, Confusion Assessment method; POD, Postoperative delirium; POCD, postoperative cognitive dysfunction; CPB, Cardiopulmonary bypass; TMT, trail making test; DST, digit substitution test; SPMSQ, Short portable mental status questionnaire; MMSE, Mini-Mental State Examination; NM, Not mentioned; ICU, Intensive care unit; SAS, Sedation agitation scale; CAM-ICU, Confusion Assessment method-intensive care unit; SD, standard deviation.

Some studies suggested that ketamine might have a protective effect against cognitive impairment. Hudetz et al. conducted two separate randomized controlled trials, both of which indicated a potential benefit of ketamine. One study found that the incidence of POD was significantly lower in the ketamine group (1/29) compared with the placebo group (9/29; P = 0.01), suggesting that ketamine attenuates POD. Another study by the same group reported that ketamine administration resulted in significantly better cognitive performance one week postoperatively compared to placebo (P < 0.01) (29, 30). Oriby et al. also observed a significant reduction in POCD in elderly patients undergoing cataract surgery under peribulbar anesthesia when ketamine was administered intravenously (P < 0.0001) (31). Similarly, Zhang et al. (2013) demonstrated a lower incidence of early POCD in elderly patients undergoing orthopedic surgery when ketamine was used (P < 0.05) (32). In another study by Soltanzadeh et al. (33), it has been demonstrated that ketamine reduced agitation in elderly patients up to 72 hours after cardiac surgery. Although the effect in the first 24 hours was not statistically significant, the long-term reduction was meaningful (P < 0.05).

On the other hand, several studies reported no significant differences in the incidence of POCD or POD when comparing ketamine with alternative anesthetic agents or placebo. For instance, Wittwer et al. conducted a prospective randomized study in elderly patients undergoing complex cardiac surgery. The study found no significant difference in the incidence of POD between the ketamine and propofol groups (P = 0.23), concluding that ketamine neither increased nor decreased cognitive dysfunction postoperatively (34). Similarly, Avidan et al. conducted a large multicenter randomized trial, examining different doses of ketamine (0.5 mg/kg and 1.0 mg/kg) compared to placebo. The results indicated no significant differences in the time to delirium onset, duration, or severity of POD (P = 0.80). The study further reported that an increase in ketamine dose was associated with a higher occurrence of hallucinations (P = 0.01) and nightmares (P = 0.03), but there was no effect on postoperative pain or opioid consumption (27). In orthopedic surgery, Lee et al. (2015) found no significant differences in cognitive function tests (Mini-Mental Status Examination, Trail-Making Test, and Digit Substitution Test) between ketamine and control groups. The study concluded that ketamine had no negative or positive effects on POCD (35).

Some studies yielded ambiguous results. Siripoonyothai et al. found that the risk of POD was higher in the propofol group compared to the ketamine group, but the effect of ketamine on POD occurrence within the first 24 hours remained unclear (P = 0.04) (36). Similarly, Rascón-Martínez et al. (2016) reported an increased number of patients with normal cognitive performance in the ketamine group postoperatively (84.8% vs. 75% in the control group, P = 0.03), but the clinical significance of this finding was debatable (37).

Several hypotheses have been proposed to explain the potential neuroprotective effects of ketamine. One mechanism suggests that ketamine, as an N-methyl-D-aspartate (NMDA) receptor antagonist, may prevent excitotoxicity caused by excessive glutamate release during surgery, thereby reducing neuroinflammation and neuronal apoptosis. This effect could explain the findings from Hudetz et al., Zhang et al., and Oriby et al., where ketamine appeared to reduce the incidence of POCD and POD (29–32). Another proposed mechanism involves ketamine’s anti-inflammatory properties. Surgical stress induces systemic inflammation, which has been linked to cognitive decline. By attenuating pro-inflammatory cytokine release, ketamine may contribute to better postoperative cognitive outcomes. It has been shown that ketamine inhibits pro-inflammatory cytokines such as interleukin-6 (IL-6) and tumor necrosis factor-alpha (TNF-α), which have been linked with cognitive impairment (38). Moreover, through the augmentation of brain-derived neurotrophic factor (BDNF) levels, ketamine may enhance cognitive resilience in elderly patients (39). Along with ketamine’s direct effect on cognitive function, it also has some indirect effects, considering its analgesic effect, which decreases opioid usage, minimizing opioid-induced cognitive impairment (40).

According to a previous meta-analysis, the incidence of POD did not differ between groups (ketamine and control) among 4 trials (RR 0.83, 95% CI [0.25, 2.80]), but patients receiving ketamine seemed at lower risk of POCD among 3 trials (RR 0.34, 95% CI [0.15, 0.73]). While their analysis provided a comprehensive view up to 2018, considering the relatively low quality of included studies, the effect of ketamine on POCD and POD needs further investigations. Moreover, this review included the adult population (≥ 18 years), which highlights the need for specific investigations in elderly patients (23).

According to available data, low-dose ketamine has been demonstrated by some research to exert a neuroprotective effect, particularly when used as an adjuvant to multimodal anesthesia. As noted above, these results confirm the hypothesized mechanisms, including reduction of neuroinflammation, enhanced synaptic plasticity, and decreased opioid use.

Despite its potential benefits, ketamine is associated with psychotomimetic side effects such as hallucinations and nightmares. Avidan et al. found a dose-dependent increase in hallucinations (P = 0.01) and nightmares (P = 0.03) in patients receiving ketamine (27). These effects could limit its widespread use in elderly patients, who may already be at higher risk of postoperative neuropsychiatric complications. These findings raised concerns about ketamine’s potential to induce cognitive dysfunction. It is hypothesized that cognitive impairment is caused by repeated dosing or by use of high-dose ketamine due to disruption of normal NMDA receptor function, increased risk of neurotoxicity, and hallucinogenic and dissociative effects. Disruption of normal NMDA receptor function would result in memory and learning deficits (41). On the other hand, excessive NMDA blockade would increase the risk of neurotoxicity (42). Furthermore, hallucinogenic and dissociative effects may interfere with cognitive recovery in older patients (43).

The discrepancies in study findings may stem from differences in study design, patient populations, surgical procedures, and cognitive assessment tools. The studies included patients aged older than 55 years, with varying levels of preoperative cognitive function and comorbidities. In addition, studies involved a range of surgeries, including cardiac, orthopedic, ophthalmic, and general surgeries. The complexity and duration of the procedures could influence cognitive outcomes. Moreover, the studies administered ketamine at different doses (0.3–1.0 mg/kg) and at varying time points (induction, intraoperative, postoperative). This variability could contribute to differing results. And last but not least, various screening instruments were used, including the Confusion Assessment Method (CAM), CAM-ICU, Mini-Mental Status Examination (MMSE), and the Intensive Care Delirium Screening Checklist, making direct comparisons challenging.

## 11 Clinical implications and future directions

Whereas current research shines a light on ketamine’s role in geriatric anesthesia, some areas in the research still need to be addressed. Given the mixed findings, further large studies are warranted to clarify ketamine’s role in preventing POCD and POD in elderly patients. Based on the available data, low-dose regimens (0.15–0.5 mg/kg IV) appear to balance cognitive benefits and minimize adverse effects in elderly patients. More well-conducted, multicenter RCTs are needed to affirm ketamine’s protective effect on older patients. In addition, preoperative cognitive screening might help identify patients at higher risk for ketamine-related cognitive impairment. On the other hand, biomarkers (e.g., inflammatory markers, BDNF levels) must be found in future research that will predict which patients will benefit most from ketamine. Future studies should focus on 1) determining the optimal dose and timing of ketamine administration to maximize its neuroprotective benefits while minimizing adverse effects, 2) investigating whether ketamine is particularly beneficial in specific subgroups of elderly patients, such as those with preexisting cognitive impairment or high inflammatory burden. Moreover, most available studies assessed cognitive function only in the early postoperative period. Since repeated ketamine exposure has been linked to persistent cognitive deficits in animal models (26, 44), long-term follow-up studies are needed to evaluate whether ketamine influences cognitive trajectory months or years after surgery. Moreover, neuroimaging studies in chronic ketamine users have shown alterations in gray matter volume and synaptic connectivity, raising concerns about long-term neurotoxicity (45). Furthermore, given the mixed cognitive effects of ketamine, researchers should explore multimodal anesthesia strategies to maximize benefits while minimizing risks in geriatrics. For example, low-dose ketamine combined with other anesthetics might reduce opioid use and inflammation while maintaining hemodynamic stability. Moreover, ketamine as an adjunct to regional anesthesia should be investigated in future clinical trials in elderly patients. Predictive analytics and artificial intelligence can be applied to tailor ketamine dosing according to genetic and metabolic phenotypes.

## 12 Conclusion

The impact of ketamine on cognitive outcomes in elderly patients remains inconclusive. It is a double-edged sword in geriatric anesthesia. While some studies report a protective effect against POCD and POD, others find no significant differences compared to placebo or alternative anesthetic agents. The variability in study methodologies, patient populations, and ketamine administration protocols likely contributes to these discrepancies. Future research should focus on standardizing these variables and exploring long-term cognitive effects to establish ketamine’s definitive role in geriatric anesthesia. In addition, future research must involve large-scale clinical trials, biomarker discovery, and personalized anesthesia protocols to specifically address the role of ketamine in maintaining cognitive function in elderly surgical patients.

## Supplementary material

Supplementary Table 1 Keywords and their alternatives used for search in databases.

Supplementary Table 2 Search strategy and related results in each database.

## References

[1] Orser BA Pennefather PS MacDonald JF. Multiple mechanisms of ketamine blockade of N-methyl-D-aspartate receptors. Anesthesiology. (1997) 86:903–17. doi: 10.1097/00000542-199704000-00021, PMID: 9105235

[2] Banik S Madavi S. Exploring the role of ketamine sedation in critically ill patients: A comprehensive review. Cureus. (2024) 16:e65836. doi: 10.7759/cureus.65836, PMID: 39219957 PMC11364493

[3] Umholtz M Nader ND. Postoperative delirium and postoperative cognitive dysfunction. In: General Anesthesia Research. Washington, DC: ASA Publications (2020). p. 239–53. doi: 10.1097/ALN.0000000000002729, PMID:

[4] Jahromi SA Parhizkar A Mohammadi M Kazemi D Tajik MH Nazari M. A comprehensive investigation of the associations between tensor-based morphometry indices and executive functions, memory, language, and visuospatial abilities in patients in the Alzheimer's disease continuum. Clin Neurol Neurosurgery. (2024) 246:108542. doi: 10.1016/j.clineuro.2024.108542, PMID: 39303664

[5] Sun J Du X Chen Y. Current progress on postoperative cognitive dysfunction: an update. J Integr Neurosci. (2024) 23:224. doi: 10.31083/j.jin2312224, PMID: 39735960

[6] Zhang MW Ho RC. Controversies of the effect of ketamine on cognition. Front Psychiatry. (2016) 7:47. doi: 10.3389/fpsyt.2016.00047, PMID: 27065891 PMC4809869

[7] Imre G Fokkema DS Den Boer JA Ter Horst GJ. Dose–response characteristics of ketamine effect on locomotion, cognitive function and central neuronal activity. Brain Res bulletin. (2006) 69:338–45. doi: 10.1016/j.brainresbull.2006.01.010, PMID: 16564431

[8] Mion G. History of anaesthesia: The ketamine story–past, present and future. Eur J Anaesthesiology. (2017) 34:571–5. doi: 10.1097/EJA.0000000000000638, PMID: 28731926

[9] Lodge D Mercier MS. Ketamine and phencyclidine: the good, the bad and the unexpected. Br J Pharmacol. (2015) 172:4254–76. doi: 10.1111/bph.13222, PMID: 26075331 PMC4556466

[10] Aroni F Iacovidou N Dontas I Pourzitaki C Xanthos T. Pharmacological aspects and potential new clinical applications of ketamine: reevaluation of an old drug. J Clin Pharmacol. (2009) 49:957–64. doi: 10.1177/0091270009337941, PMID: 19546251

[11] Sneyd J. Recent advances in intravenous anaesthesia. Br J anaesthesia. (2004) 93:725–36. doi: 10.1093/bja/aeh253, PMID: 15347606

[12] Bredmose PP Grier G Davies GE Lockey DJ. Pre-hospital use of ketamine in paediatric trauma. Acta Anaesthesiol Scand. (2009) 53:543–5. doi: 10.1111/j.1399-6576.2008.01852.x, PMID: 19226295

[13] Roelofse JA. The evolution of ketamine applications in children. Pediatr Anesthesia. (2010) 20:240–5. doi: 10.1111/j.1460-9592.2009.03145.x, PMID: 19793346

[14] Sleigh J Harvey M Voss L Denny B. Ketamine–More mechanisms of action than just NMDA blockade. Trends Anaesthesia Crit Care. (2014) 4:76–81. doi: 10.1016/j.tacc.2014.03.002

[15] Choudhury D Autry AE Tolias KF Krishnan V. Ketamine: neuroprotective or neurotoxic? Front Neurosci. (2021) 15:672526. doi: 10.3389/fnins.2021.672526, PMID: 34566558 PMC8461018

[16] Haile C Murrough J Iosifescu D Chang L Al Jurdi R Foulkes A. Plasma brain derived neurotrophic factor (BDNF) and response to ketamine in treatment-resistant depression. Int J Neuropsychopharmacol. (2014) 17:331–6. doi: 10.1017/S1461145713001119, PMID: 24103211 PMC3992942

[17] Loveday BA Sindt J. Ketamine protocol for palliative care in cancer patients with refractory pain. J Adv Practitioner Oncol. (2015) 6:555–61. doi: 10.6004/jadpro.6.6.4, PMID: 27648345 PMC5017546

[18] Vlisides P Xie Z. Neurotoxicity of general anesthetics: an update. Curr Pharm Design. (2012) 18:6232–40. doi: 10.2174/138161212803832344, PMID: 22762477

[19] Kim SH Im Kim S Ok SY Park SY Kim M-G Lee S-J. Opioid sparing effect of low dose ketamine in patients with intravenous patient-controlled analgesia using fentanyl after lumbar spinal fusion surgery. Korean J Anesthesiology. (2013) 64:524–8. doi: 10.4097/kjae.2013.64.6.524, PMID: 23814653 PMC3695250

[20] Kamp J Olofsen E Henthorn TK Van Velzen M Niesters M Dahan A. Ketamine pharmacokinetics: a systematic review of the literature, meta-analysis, and population analysis. Anesthesiology. (2020) 133:1192–213. doi: 10.1097/ALN.0000000000003577, PMID: 32997732

[21] Eilers H Niemann CU. Clinically important drug interactions with intravenous anaesthetics in older patients. Drugs Aging. (2003) 20:969–80. doi: 10.2165/00002512-200320130-00002, PMID: 14561101

[22] Peltoniemi MA Hagelberg NM Olkkola KT Saari TI. Ketamine: a review of clinical pharmacokinetics and pharmacodynamics in anesthesia and pain therapy. Clin pharmacokinetics. (2016) 55:1059–77. doi: 10.1007/s40262-016-0383-6, PMID: 27028535

[23] Hovaguimian F Tschopp C Beck-Schimmer B Puhan M. Intraoperative ketamine administration to prevent delirium or postoperative cognitive dysfunction: A systematic review and meta-analysis. Acta Anaesthesiologica Scandinavica. (2018) 62:1182–93. doi: 10.1111/aas.2018.62.issue-9 29947091

[24] Hudetz JA Pagel PS. Neuroprotection by ketamine: a review of the experimental and clinical evidence. J cardiothoracic Vasc anesthesia. (2010) 24:131–42. doi: 10.1053/j.jvca.2009.05.008, PMID: 19640746

[25] Mohammad Shehata I Masood W Nemr N Anderson A Bhusal K Edinoff AN. The possible application of ketamine in the treatment of depression in Alzheimer’s disease. Neurol Int. (2022) 14:310–21. doi: 10.3390/neurolint14020025, PMID: 35466206 PMC9036213

[26] Luo Y Yu Y Zhang M He H Fan N. Chronic administration of ketamine induces cognitive deterioration by restraining synaptic signaling. Mol Psychiatry. (2021) 26:4702–18. doi: 10.1038/s41380-020-0793-6, PMID: 32488127

[27] Avidan MS Maybrier HR Abdallah AB Jacobsohn E Vlisides PE Pryor KO. Intraoperative ketamine for prevention of postoperative delirium or pain after major surgery in older adults: an international, multicentre, double-blind, randomised clinical trial. Lancet. (2017) 390:267–75. doi: 10.1016/S0140-6736(17)31467-8, PMID: 28576285 PMC5644286

[28] Loo CK Katalinic N Garfield JB Sainsbury K Hadzi-Pavlovic D Mac-Pherson R. Neuropsychological and mood effects of ketamine in electroconvulsive therapy: a randomised controlled trial. J Affect Disord. (2012) 142:233–40. doi: 10.1016/j.jad.2012.04.032, PMID: 22858219

[29] Hudetz J Iqbal Z Gandhi S Patterson K Byrne A Hudetz A. Ketamine attenuates post-operative cognitive dysfunction after cardiac surgery. Acta Anaesthesiologica Scandinavica. (2009) 53:864–72. doi: 10.1111/j.1399-6576.2009.01978.x, PMID: 19422355

[30] Hudetz JA Patterson KM Iqbal Z Gandhi SD Byrne AJ Hudetz AG. Ketamine attenuates delirium after cardiac surgery with cardiopulmonary bypass. J Cardiothoracic Vasc Anesthesia. (2009) 23:651–7. doi: 10.1053/j.jvca.2008.12.021, PMID: 19231245

[31] Oriby ME Elrashidy AA Elsharkawy A Ahmed SA. Effects of ketamine or dexmedetomidine on postoperative cognitive dysfunction after cataract surgery: a randomized controlled trial. Indian J Anaesthesia. (2023) 67:186–93. doi: 10.4103/ija.ija_429_22, PMID: 37091455 PMC10121090

[32] Zhang X Piao M Wang Y Feng C. Influence of sub-anesthetic dose of ketamine and dexmedetomidine on early postoperative cognitive function in elderly orthopedic patients under total intravenous anesthesia. J Jilin Univ (Medicine Edition). (2013) 39:133–7. doi: 10.7694/jldxyxb20130130

[33] Soltanzadeh M Ebadi A Firoozabadi MD Tabatabee S Babaee A. The effect of intravenous ketamine during cardiopulmonary bypass on postoperative agitation. Iranian J Psychiatry Clin Psychol. (2013) 2:24–31.

[34] Wittwer ED Cerhan JH Schroeder DR Schaff HV Mauermann WJ. Impact of ketamine versus propofol for anesthetic induction on cognitive dysfunction, delirium, and acute kidney injury following cardiac surgery in elderly, high-risk patients. Ann cardiac anaesthesia. (2023) 26:274–80. doi: 10.4103/aca.aca_106_22, PMID: 37470525 PMC10451121

[35] Lee KH Kim JY Kim JW Park JS Lee KW Jeon SY. Influence of ketamine on early postoperative cognitive function after orthopedic surgery in elderly patients. Anesthesiology Pain Med. (2015) 5:e28844. doi: 10.5812/aapm.28844, PMID: 26587403 PMC4644306

[36] Siripoonyothai S Sindhvananda W. Comparison of postoperative delirium within 24 hours between ketamine and propofol infusion during cardiopulmonary bypass machine: a randomized controlled trial. Ann Cardiac Anaesthesia. (2021) 24:294–301. doi: 10.4103/aca.ACA_85_20, PMID: 34269257 PMC8404598

[37] Rascón-Martínez DM Fresán-Orellana A Ocharán-Hernández ME Genis-Zarate JH Castellanos-Olivares A. The effects of ketamine on cognitive function in elderly patients undergoing ophthalmic surgery: a pilot study. Anesth Analgesia. (2016) 122:969–75. doi: 10.1213/ANE.0000000000001153, PMID: 26771268

[38] Wang N Yu H-Y Shen X-F Gao Z-Q Yang C Yang J-J. The rapid antidepressant effect of ketamine in rats is associated with down-regulation of pro-inflammatory cytokines in the hippocampus. Upsala J Med Sci. (2015) 120:241–8. doi: 10.3109/03009734.2015.1060281, PMID: 26220286 PMC4816884

[39] Ricci V Martinotti G Gelfo F Tonioni F Caltagirone C Bria P. Chronic ketamine use increases serum levels of brain-derived neurotrophic factor. Psychopharmacology. (2011) 215:143–8. doi: 10.1007/s00213-010-2121-3, PMID: 21161184

[40] Kissin I Bright CA Bradley EL Jr. The effect of ketamine on opioid-induced acute tolerance: can it explain reduction of opioid consumption with ketamine-opioid analgesic combinations? Anesth Analgesia. (2000) 91:1483–8. doi: 10.1097/00000539-200012000-00035, PMID: 11094005

[41] Newcomer JW Krystal JH. NMDA receptor regulation of memory and behavior in humans. Hippocampus. (2001) 11:529–42. doi: 10.1002/hipo.v11:5, PMID: 11732706

[42] Lipton SA. Paradigm shift in neuroprotection by NMDA receptor blockade: memantine and beyond. Nat Rev Drug Discov. (2006) 5:160–70. doi: 10.1038/nrd1958, PMID: 16424917

[43] Denomme N Heifets BD. Ketamine, the first associative anesthetic? some considerations on classifying psychedelics, entactogens, and dissociatives. Washington, DC: American Psychiatric Association (2024) p. 784–6. 10.1176/appi.ajp.20240644 39217435

[44] Ding R Li Y Du A Yu H He B Shen R. Changes in hippocampal AMPA receptors and cognitive impairments in chronic ketamine addiction models: another understanding of ketamine CNS toxicity. Sci Rep. (2016) 6:38771. doi: 10.1038/srep38771, PMID: 27934938 PMC5146946

[45] Strous JF Weeland CJ van der Draai FA Daams JG Denys D Lok A. Brain changes associated with long-term ketamine abuse, a systematic review. Front Neuroanatomy. (2022) 16:795231. doi: 10.3389/fnana.2022.795231, PMID: 35370568 PMC8972190

