# Supplementary material for: The impact of ketamine on cognitive outcomes in geriatric anesthesia: a comprehensive review
Source: Front Psychiatry. 2025 Jul 30;16:1594730. doi: 10.3389/fpsyt.2025.1594730 (PMC12343599; doi:10.3389/fpsyt.2025.1594730)
Supplement: Supplementary Table 1 — Keywords and their alternatives used for search in databases. [file Table1.docx]

**Supplementary**

| Table S1- Keywords and their alternatives used for search in databases | | | |
| --- | --- | --- | --- |
| Ketamine | Cognitive function | Anesthesia | Old population |
| ketamine  2-(2-Chlorophenyl)-2-(methylamino)cyclohexanone  Ketalar  CI581  CI-581  CI 581  Ketanest  Ketamine Hydrochloride  Calipsol  Calypsol  Kalipsol  Ketaset  Esketamine | Cognit*  Neurocognit*  Cognit* Function*  Cognit* Impairment*  Cognit* Decline*  Cognit* Performance*  Cognit* Measure*  Cognit* Dysfunction*  Cognit* Deterioration*  Cognit* Development*  Cognit* Deficit*  Cognit* Disorder  Mental Deterioration  Delirium*  postoperative cognitive dysfunction  postoperative delirium  postoperative cognitive complications | Anesthe*  Anaesth*  Surger*  Operat* | Elderly  old  aged  Geriatric*  Gerontology |

| Table S2- Search strategy and related results in each database | | |
| --- | --- | --- |
| Database | Search strategy | Results |
| Scopus | #1  ( TITLE-ABS-KEY ( ketamine ) OR TITLE-ABS-KEY ( 2 2 chlorophenyl 2 methylamino AND cyclohexanone ) OR TITLE-ABS-KEY ( ketalar ) OR TITLE-ABS-KEY ( ci581 ) OR TITLE-ABS-KEY ( ci-581 ) OR TITLE-ABS-KEY ( ci 581 ) OR TITLE-ABS-KEY ( ketanest ) OR TITLE-ABS-KEY ( ketamine AND hydrochloride ) OR TITLE-ABS-KEY ( calipsol ) OR TITLE-ABS-KEY ( calypsol ) OR TITLE-ABS-KEY ( kalipsol ) OR TITLE-ABS-KEY ( ketaset ) OR TITLE-ABS-KEY ( esketamine ) ) | 73,653 |
|  | #2  ( TITLE-ABS-KEY ( cognit* ) OR TITLE-ABS-KEY ( neurocognit* ) OR TITLE-ABS-KEY ( cognit* AND function* ) OR TITLE-ABS-KEY ( cognit* AND impairment* ) OR TITLE-ABS-KEY ( cognit* AND decline* ) OR TITLE-ABS-KEY ( cognit* AND performance* ) OR TITLE-ABS-KEY ( cognit* AND measure* ) OR TITLE-ABS-KEY ( cognit* AND dysfunction* ) OR TITLE-ABS-KEY ( cognit* AND deterioration* ) OR TITLE-ABS-KEY ( cognit* AND development* ) OR TITLE-ABS-KEY ( cognit* AND deficit* ) OR TITLE-ABS-KEY ( cognit* AND disorder ) OR TITLE-ABS-KEY ( mental AND deterioration ) OR TITLE-ABS-KEY ( delirium* ) OR TITLE-ABS-KEY ( postoperative AND cognitive AND dysfunction ) OR TITLE-ABS-KEY ( postoperative AND delirium ) OR TITLE-ABS-KEY ( postoperative AND cognitive AND complications ) ) | 1,415,582 |
|  | #3  ( TITLE-ABS-KEY ( anesthe* ) OR TITLE-ABS-KEY ( anaesth* ) OR TITLE-ABS-KEY ( surger* ) OR TITLE-ABS-KEY ( operat* ) ) | 9,738,471 |
|  | #4  ( TITLE-ABS-KEY ( elderly ) OR TITLE-ABS-KEY ( old ) OR TITLE-ABS-KEY ( aged ) OR TITLE-ABS-KEY ( geriatric* ) OR TITLE-ABS-KEY ( gerontology ) ) | 8,667,978 |
|  | #1 AND #2 AND #3 AND #4 | 648 |
| Web of Science | #1  ketamine (Topic) or 2-(2-Chlorophenyl)-2-(methylamino)cyclohexanone (Topic) or Ketalar (Topic) or CI581 (Topic) or CI-581 (Topic) or CI 581 (Topic) or Ketanest (Topic) or Ketamine Hydrochloride (Topic) or Calipsol (Topic) or Calypsol (Topic) or Kalipsol (Topic) or Ketaset (Topic) or Esketamine (Topic) | [35,954](https://www.webofscience.com/wos/woscc/summary/1c1f0d5c-5301-4821-99e2-0ef9e7e27a25-016551de3b/relevance/1) |
|  | #2  Cognit* (Topic) or Neurocognit* (Topic) or Cognit* Function* (Topic) or Cognit* Impairment* (Topic) or Cognit* Decline* (Topic) or Cognit* Performance* (Topic) or Cognit* Measure* (Topic) or Cognit* Dysfunction* (Topic) or Cognit* Deterioration* (Topic) or Cognit* Development* (Topic) or Cognit* Deficit* (Topic) or Cognit* Disorder (Topic) or Mental Deterioration (Topic) or Delirium* (Topic) or postoperative cognitive dysfunction (Topic) or Postoperative delirium (Topic) or postoperative cognitive complications (Topic) | [1,174,397](https://www.webofscience.com/wos/woscc/summary/551afb29-7292-41df-b8ed-b41308311090-0165523c00/relevance/1) |
|  | #3  Anesthe* (Topic) or Anaesth* (Topic) or Surger* (Topic) or Operat* (Topic) | [6,107,831](https://www.webofscience.com/wos/woscc/summary/230bac04-bde3-46b5-a573-ca914073f8b3-0165526567/relevance/1) |
|  | #4  Elderly (Topic) or old (Topic) or aged (Topic) or Geriatric* (Topic) or Gerontology (Topic) | [6,689,595](https://www.webofscience.com/wos/woscc/summary/c48e8722-6b4c-441e-8ed7-fbcb055a2633-0165528cfd/relevance/1) |
|  | #1 AND #2 AND #3 AND #4 | [366](https://www.webofscience.com/wos/woscc/summary/d73fcbe4-610b-49a2-9dfc-c354d32d5648-016552a24b/relevance/1) |
| PubMed | #1  ((((((((((((ketamine[Title/Abstract]) OR (2-(2-Chlorophenyl)-2-(methylamino)cyclohexanone[Title/Abstract])) OR (Ketalar[Title/Abstract])) OR (CI581[Title/Abstract])) OR (CI-581[Title/Abstract])) OR (CI 581[Title/Abstract])) OR (Ketanest[Title/Abstract])) OR (Ketamine Hydrochloride[Title/Abstract])) OR (Calipsol[Title/Abstract])) OR (Calypsol[Title/Abstract])) OR (Kalipsol[Title/Abstract])) OR (Ketaset[Title/Abstract])) OR (Esketamine[Title/Abstract]) | [25,731](https://pubmed.ncbi.nlm.nih.gov/?term=%28%28%28%28%28%28%28%28%28%28%28%28ketamine%5BTitle%2FAbstract%5D%29+OR+%282-%282-Chlorophenyl%29-2-%28methylamino%29cyclohexanone%5BTitle%2FAbstract%5D%29%29+OR+%28Ketalar%5BTitle%2FAbstract%5D%29%29+OR+%28CI581%5BTitle%2FAbstract%5D%29%29+OR+%28CI-581%5BTitle%2FAbstract%5D%29%29+OR+%28CI+581%5BTitle%2FAbstract%5D%29%29+OR+%28Ketanest%5BTitle%2FAbstract%5D%29%29+OR+%28Ketamine+Hydrochloride%5BTitle%2FAbstract%5D%29%29+OR+%28Calipsol%5BTitle%2FAbstract%5D%29%29+OR+%28Calypsol%5BTitle%2FAbstract%5D%29%29+OR+%28Kalipsol%5BTitle%2FAbstract%5D%29%29+OR+%28Ketaset%5BTitle%2FAbstract%5D%29%29+OR+%28Esketamine%5BTitle%2FAbstract%5D%29&sort=) |
|  | #2  ((((((((((((((((Cognit*[Title/Abstract]) OR (Neurocognit*[Title/Abstract])) OR (Cognit* Function*[Title/Abstract])) OR (Cognit* Impairment*[Title/Abstract])) OR (Cognit* Decline*[Title/Abstract])) OR (Cognit* Performance*[Title/Abstract])) OR (Cognit* Measure*[Title/Abstract])) OR (Cognit* Dysfunction*[Title/Abstract])) OR (Cognit* Deterioration*[Title/Abstract])) OR (Cognit* Development*[Title/Abstract])) OR (Cognit* Deficit*[Title/Abstract])) OR (Cognit* Disorder[Title/Abstract])) OR (Mental Deterioration[Title/Abstract])) OR (Delirium*[Title/Abstract])) OR (postoperative cognitive dysfunction[Title/Abstract])) OR (Postoperative delirium[Title/Abstract])) OR (postoperative cognitive complications[Title/Abstract]) | [651,003](https://pubmed.ncbi.nlm.nih.gov/?term=%28%28%28%28%28%28%28%28%28%28%28%28%28%28%28%28Cognit%2A%5BTitle%2FAbstract%5D%29+OR+%28Neurocognit%2A%5BTitle%2FAbstract%5D%29%29+OR+%28Cognit%2A+Function%2A%5BTitle%2FAbstract%5D%29%29+OR+%28Cognit%2A+Impairment%2A%5BTitle%2FAbstract%5D%29%29+OR+%28Cognit%2A+Decline%2A%5BTitle%2FAbstract%5D%29%29+OR+%28Cognit%2A+Performance%2A%5BTitle%2FAbstract%5D%29%29+OR+%28Cognit%2A+Measure%2A%5BTitle%2FAbstract%5D%29%29+OR+%28Cognit%2A+Dysfunction%2A%5BTitle%2FAbstract%5D%29%29+OR+%28Cognit%2A+Deterioration%2A%5BTitle%2FAbstract%5D%29%29+OR+%28Cognit%2A+Development%2A%5BTitle%2FAbstract%5D%29%29+OR+%28Cognit%2A+Deficit%2A%5BTitle%2FAbstract%5D%29%29+OR+%28Cognit%2A+Disorder%5BTitle%2FAbstract%5D%29%29+OR+%28Mental+Deterioration%5BTitle%2FAbstract%5D%29%29+OR+%28Delirium%2A%5BTitle%2FAbstract%5D%29%29+OR+%28postoperative+cognitive+dysfunction%5BTitle%2FAbstract%5D%29%29+OR+%28Postoperative+delirium%5BTitle%2FAbstract%5D%29%29+OR+%28postoperative+cognitive+complications%5BTitle%2FAbstract%5D%29&sort=) |
|  | #3  (((Anesthe*[Title/Abstract]) OR (Anaesth*[Title/Abstract])) OR (Surger*[Title/Abstract])) OR (Operat*[Title/Abstract]) | [3,132,365](https://pubmed.ncbi.nlm.nih.gov/?term=%28%28%28Anesthe%2A%5BTitle%2FAbstract%5D%29+OR+%28Anaesth%2A%5BTitle%2FAbstract%5D%29%29+OR+%28Surger%2A%5BTitle%2FAbstract%5D%29%29+OR+%28Operat%2A%5BTitle%2FAbstract%5D%29&sort=) |
|  | #4  ((((Elderly[Title/Abstract]) OR (old[Title/Abstract])) OR (aged[Title/Abstract])) OR (Geriatric*[Title/Abstract])) OR (Gerontology[Title/Abstract]) | [2,411,592](https://pubmed.ncbi.nlm.nih.gov/?term=%28%28%28%28Elderly%5BTitle%2FAbstract%5D%29+OR+%28old%5BTitle%2FAbstract%5D%29%29+OR+%28aged%5BTitle%2FAbstract%5D%29%29+OR+%28Geriatric%2A%5BTitle%2FAbstract%5D%29%29+OR+%28Gerontology%5BTitle%2FAbstract%5D%29&sort=) |
|  | #1 AND #2 AND #3 AND #4 | [157](https://pubmed.ncbi.nlm.nih.gov/?term=%28%28%28%28%28%28%28%28%28%28%28%28%28%28%28ketamine%5BTitle%2FAbstract%5D%29+OR+%282-%282-Chlorophenyl%29-2-%28methylamino%29cyclohexanone%5BTitle%2FAbstract%5D%29%29+OR+%28Ketalar%5BTitle%2FAbstract%5D%29%29+OR+%28CI581%5BTitle%2FAbstract%5D%29%29+OR+%28CI-581%5BTitle%2FAbstract%5D%29%29+OR+%28CI+581%5BTitle%2FAbstract%5D%29%29+OR+%28Ketanest%5BTitle%2FAbstract%5D%29%29+OR+%28Ketamine+Hydrochloride%5BTitle%2FAbstract%5D%29%29+OR+%28Calipsol%5BTitle%2FAbstract%5D%29%29+OR+%28Calypsol%5BTitle%2FAbstract%5D%29%29+OR+%28Kalipsol%5BTitle%2FAbstract%5D%29%29+OR+%28Ketaset%5BTitle%2FAbstract%5D%29%29+OR+%28Esketamine%5BTitle%2FAbstract%5D%29%29+AND+%28%28%28%28%28%28%28%28%28%28%28%28%28%28%28%28%28Cognit%2A%5BTitle%2FAbstract%5D%29+OR+%28Neurocognit%2A%5BTitle%2FAbstract%5D%29%29+OR+%28Cognit%2A+Function%2A%5BTitle%2FAbstract%5D%29%29+OR+%28Cognit%2A+Impairment%2A%5BTitle%2FAbstract%5D%29%29+OR+%28Cognit%2A+Decline%2A%5BTitle%2FAbstract%5D%29%29+OR+%28Cognit%2A+Performance%2A%5BTitle%2FAbstract%5D%29%29+OR+%28Cognit%2A+Measure%2A%5BTitle%2FAbstract%5D%29%29+OR+%28Cognit%2A+Dysfunction%2A%5BTitle%2FAbstract%5D%29%29+OR+%28Cognit%2A+Deterioration%2A%5BTitle%2FAbstract%5D%29%29+OR+%28Cognit%2A+Development%2A%5BTitle%2FAbstract%5D%29%29+OR+%28Cognit%2A+Deficit%2A%5BTitle%2FAbstract%5D%29%29+OR+%28Cognit%2A+Disorder%5BTitle%2FAbstract%5D%29%29+OR+%28Mental+Deterioration%5BTitle%2FAbstract%5D%29%29+OR+%28Delirium%2A%5BTitle%2FAbstract%5D%29%29+OR+%28postoperative+cognitive+dysfunction%5BTitle%2FAbstract%5D%29%29+OR+%28Postoperative+delirium%5BTitle%2FAbstract%5D%29%29+OR+%28postoperative+cognitive+complications%5BTitle%2FAbstract%5D%29%29%29+AND+%28%28%28%28Anesthe%2A%5BTitle%2FAbstract%5D%29+OR+%28Anaesth%2A%5BTitle%2FAbstract%5D%29%29+OR+%28Surger%2A%5BTitle%2FAbstract%5D%29%29+OR+%28Operat%2A%5BTitle%2FAbstract%5D%29%29%29+AND+%28%28%28%28%28Elderly%5BTitle%2FAbstract%5D%29+OR+%28old%5BTitle%2FAbstract%5D%29%29+OR+%28aged%5BTitle%2FAbstract%5D%29%29+OR+%28Geriatric%2A%5BTitle%2FAbstract%5D%29%29+OR+%28Gerontology%5BTitle%2FAbstract%5D%29%29&sort=) |
